# Recent advances in pharmacological management of urinary incontinence

**DOI:** 10.12688/f1000research.12593.1

**Published:** 2017-12-19

**Authors:** Bronagh McDonnell, Lori Ann Birder

**Affiliations:** 1 Department of Medicine, University of Pittsburgh School of Medicine, A 1217 Scaife Hall, 3550 Terrace Street, Pittsburgh, PA, 15261, USA; 2 Department of Pharmacology and Chemical Biology, University of Pittsburgh School of Medicine, A 1217 Scaife Hall, 3550 Terrace Street, Pittsburgh, PA, 15261, USA

**Keywords:** urinary incontinence

## Abstract

Lower urinary tract symptoms—in particular, storage disorders (for example, urinary incontinence) as well as bladder underactivity—are major health-related problems that increase with age. Yet lower urinary tract symptoms remain under-diagnosed and poorly managed, and incontinence has been cited as the major reason for institutionalization in elderly populations and is one of the most common conditions in primary care practice. Although lifestyle and behavior therapy has been used as a useful treatment regimen for urge incontinence, medications (often used as adjunct) can provide additional benefit. This review will include current therapies used for treatment of urinary incontinence.

## Introduction

Urinary incontinence (UI), alone or as an overactive bladder (OAB) symptom, is a growing global health-care problem that increases in prevalence with age ^1– 3^. UI is 1.5 times more common in women than men, affecting approximately 45% and 77% of adult males and females, respectively, in the US, the UK, and Sweden ^4^. Both severity and frequency of UI significantly increase mortality among men and women ^5^. An industry report recently predicted a 2.9% global market growth rate of pharmacological treatments for OAB from 2017 to 2022 and projected a total cost of USD $4.19 billion by the year 2020 ^6^. Despite its widespread prevalence and socioeconomic burden, incontinence therapy remains limited and often accompanied by significant adverse effects (AEs), low efficacy, and high discontinuation rates ^7, 8^. In addition, there is surprisingly little information regarding biomarkers that can identify age-induced bladder dysfunction or patient response to treatment. There is a growing and pervasive need for pharmacologic therapies that are efficacious, safe, and tolerable. Efforts in the past three years have identified potential new targets in addition to combination therapies or adding a non-pharmacological intervention or both. This article will highlight advances within the last three years regarding current pharmacological treatments for both urge UI (UUI) and stress UI (SUI).

## UI subtypes

Clinical and basic science has classified UI into subtypes based on pathophysiology and causal factors. The most bothersome subtype reported by women is UUI ^4, 9^ whereas the most prevalent subtype is SUI ^4, 10^. The pathophysiology underlying UUI is complex and based on altered physiological mechanisms controlling bladder storage and emptying. Causal factors have been focused on myogenic, neurogenic, and (more recently) sensory dysfunction (see review 11). SUI is urinary leakage due to increased abdominal pressure caused by sneezing, coughing, exercise, lifting, and position change. Its pathophysiology, compared with that of UUI, may be less complex and largely related to physical changes in structures forming or in relation to the lower urinary tract (for example, the pelvic floor muscles). A large proportion of women experience mixed urinary incontinence (MUI), which includes both UUI and SUI symptoms. Studies have reported MUI alone to affect 2–4 out of 10 women who are from 20 to more than 80 years old ^4, 9^. Following clinical determination of urge or stress incontinence, a number of treatment regimens can be followed. Deciding on a treatment regimen is a multi-factorial decision process dictated in part by UI subtype and symptom severity.

## Pharmacological management for urge incontinence

Anti-muscarinic (anti-cholinergic) drugs remain the most widely used treatment option for UUI and often are used in primary care in combination with behavioral and lifestyle changes, which can include pelvic floor muscle training exercises or reduction of caffeine intake or both. An unfavorable balance between efficacy and tolerability of anti-muscarinic agents, despite their widespread use, exists and has raised questions regarding the overall benefits derived from use of these agents. Furthermore, caution regarding use of anti-muscarinics is recommended in frail elderly patients because of an increased risk of cognitive impairment ^12, 13^. Oxybutynin is the most widely prescribed anti-muscarinic for UUI ^14^. Other drugs, such as β3-agonists and phosphodiesterase 5 (PDE5) inhibitors, are available and may have fewer unwanted AEs but have not been shown to be any more effective in improving storage lower urinary tract symptoms. Several advances are being made to reduce anti-cholinergic side effects primarily by creating receptor-selective agents, altering formulation or administration (or both), thus minimizing AEs caused by systemic antagonism of cholinergic receptors.

Currently licensed anti-muscarinics indicated for incontinence include orally prescribed oxybutynin, tolterodine, and trospium available as immediate release (IR) and extended release (ER) formulations. In addition, US Food and Drug Administration (FDA)-approved anti-muscarinics include fesoterodine, darifenacin, and solifenacin as oral IR formulations. Propiverine is an oral anti-muscarinic approved in Asia and recently in Canada and is awaiting FDA approval for the US ^11^. Oxybutynin is the most widely available anti-muscarinic in the US, where it is also available in oral ER and transdermal formulations ^13^.

Transdermal formulations of oxybutynin and tolterodine were introduced in 2007 to avoid/minimize systemic AEs associated with oral treatments. A comparison study in 2017 involving 1,530 patients (more than 20 years old) with OAB assessed efficacy and toxicity at 12 weeks between the oxybutynin patch and oral propiverine (20 mg). The authors reported non-inferiority in terms of reducing the number of incontinence episodes (urge or total incontinence). While a greater incidence of AEs was reported with oxybutynin patch versus propiverine group (74.8% versus 64.9%), the incidence of dry mouth or constipation was reduced more than 50% compared with propiverine. The predominant AE associated with the patch, which affected 40.2% of patients, was dermatitis and was reportedly mild in the majority of cases; 26.7% of the affected group (10.7% total) continued treatment with topical steroidal treatment ^15^. A phase 3 study assessed the efficacy and safety of once-daily applied oxybutynin transdermal gel 3% (OTG3%) (84 or 56 mg) or placebo in 624 adult patients with OAB-UUI. At 12 weeks, OTG3% 84 mg reduced the weekly number of incontinence episodes (urge and mixed) compared with placebo (difference from baseline, -20.4 versus -18.1, respectively). The study concluded that OTG3% 84 mg/day was well tolerated and effective in this adult population ^16^. Lastly, a large meta-analysis in women with OAB found that long-acting oxybutynin formulations (oral, patch, and gel), compared with IR, were more effective, had a lower incidence of AEs, and were better tolerated ^17^.

Little evidence is available to support the long-term use of anti-muscarinics. Besides the typical systemic effects which can include dry mouth, constipation, and blurry vision, increasing new evidence indicates that cumulative anti-muscarinic use is associated with an increase in incident dementia ^18^ as well as mood disorders, including depression ^19^. These findings support earlier reports of associated depression with short-term anti-muscarinic treatment ^20^. The new knowledge of incident dementia and mood disorders warrants careful consideration by clinicians and patients when considering treatment options for incontinence, particularly in elderly patients and those with neurobiological disease such as multiple sclerosis and Alzheimer’s disease. In the past three years, clinicians have created FORTA (Fit fOR The Aged), a clinical tool to help aid decision making regarding appropriateness of pharmacotherapies in elderly patients often being treated for multiple conditions ^21^.

### Children

Anti-muscarinics are the main pharmacological treatment for children (<12 years) when non-pharmacological or neuromodulation or both are not effective, usually in severe cases ^22^. Oxybutynin IR and ER (dosing 0.3–0.6 mg/kg per day daily dose, not exceeding 15 mg/day total) are the only currently FDA-approved anti-muscarinics for treatment of OAB symptoms in children. Newer anti-muscarinics have not yet been approved but are prescribed off-label in treatment-resistant children. In these cases, tolterodine and solifenacin have shown comparable efficacy with fewer side effects ^22^. Propiverine (oral) (0.4–0.8mg/kg per day in two doses) is approved for children in other countries, including Japan ^23^. Trials in children have reported efficacy in reducing incontinence with an acceptable tolerability using propiverine open-label ^24, 25^ and trospium ^26^. Oxybutynin transdermal patches may offer a viable alternative to oral oxybutynin, reducing AEs and difficulty of dosing, in children with OAB symptoms, including incontinence ^27^. Solifenacin seems a promising drug option for children and adolescents with incontinence as part of OAB and non-neurogenic detrusor overactivity and those refractory to other anti-muscarinics (oxybutynin and tolterodine treatment). Nadeau *et al*. have reported efficacy using solifenacin in children as part of a dual anti-muscarinic therapy approach ^28^. In addition, a phase 3 placebo-controlled trial concluded a clinically relevant improvement in mean voided volume in children at 12 weeks following once-daily treatment with solifenacin oral suspension ^29^. This study was one of the first to report long-term use of solifenacin in 119 children/adolescents compared with placebo, reporting improvement in incontinence by 3 weeks (change from baseline) which was maintained for the study period (52 weeks) ^30^. Further studies are needed to establish efficacy, safety, and tolerability compared with placebo and other anti-muscarinics.

### Beta-adrenergic receptor agonists

Mirabegron, a β3-adrenoceptor (AR) agonist, is a first-in-its-class drug approved for the treatment of OAB with symptoms of UUI, urgency, and urinary frequency ^31, 32^. This drug is associated with improved bladder compliance, increased bladder capacity, reduced urinary frequency, and reduced incontinence ^32^. Recent evidence has suggested that mirabegron may relax the smooth muscle via a dual mechanism of action which includes activation of β3-AR in addition to blockade α1-AR ^33, 34^. It is acknowledged that long-term toxicity and efficacy, compared with those of standard treatment, remain to be established.

In phase II and III clinical trials, mirabegron was found to be efficacious in reducing OAB symptoms as well as reducing the number of incontinence episodes. Improvement was found to be maintained throughout treatment (up to 12 months) and appeared to be well tolerated in patients, including those at least 65 years old ^35, 36^. A recent *post hoc* analysis of three phase III randomized control trials (RCTs) in women with OAB-incontinence concluded a significant improvement in the number of incontinence episodes with mirabegron versus placebo and established a greater treatment effect with increasing severity of incontinence ^37^. These results raise the possibility that mirabegron is more effective in severe forms of OAB-incontinence and thus may provide a preferred treatment option for these patients. Available evidence would suggest that it offers an effective alternative to anti-muscarinic therapy ^38^.

A large meta-analysis of 44 RCTs of 27,309 patients concluded that mirabegron (50 mg) has similar efficacy to most anti-muscarinic drugs but with fewer incidences of AEs, particularly dry mouth, which is the most frequently reported anti-muscarinic AE and reason for discontinuation among patients ^39^. Further head-to-head comparisons between mirabegron and anti-muscarinic drugs are necessary to compare efficacy and safety. Notably, mirabegron appears effective in adult patients who are refractory to or unsuitable for anti-muscarinic therapy ^35^. This finding is further supported by a recent phase III, multicenter, open-label RCT in Japan, where the authors reported long-term (52 weeks) efficacy and safety of mirabegron in patients with OAB. The majority of patients remained on 50 mg treatment with concomitant improvement in all domains of quality of life (QOL) scores ^40^.

Mirabegron is not currently licensed for use in children; however, it is prescribed open-label for various types of incontinence, including nocturnal enuresis and daytime incontinence. Safety and efficacy in children remain to be confirmed. Two open-label trials in Canada have shown mirabegron to be effective and safe in children (ages 5 to 17 years) with OAB-incontinence/enuresis ^41, 42^. Blais *et al*. (2016) reported improvement in continence and QOL in children/adolescents with refractory OAB and unsatisfactory management of symptoms with anti-muscarinic therapy ^41^. The second trial reported mirabegron to be beneficial as an add-on therapy along with anti-muscarinic therapy, again in children with refractory OAB. The addition of mirabegron to the treatment schedule was well tolerated and appeared to be safe ^42^.

### Combination therapy

Combination therapy using an anti-cholinergic agent coupled with mirabegron has also been used. Several phase III trials in the past three years have assessed its efficacy and safety as add-on to anti-muscarinic treatment (dual therapy). The phase II randomized control SYMPHONY trial (2015) assessed combination mirabegron 25 or 50 mg and solifenacin 5 or 10 mg in women with OAB ^43^. In a follow-up study, SYMPHONY II, Abrams *et al*. reported improvement in objective and subjective efficacy outcomes with combination mirabegron 25/50 mg and solifenacin 5/10 mg compared with placebo or solifenacin 5 mg alone ^44^. Herschorn *et al*. evaluated solifenacin 5 mg combined with mirabegron 25 or 50 mg and found superior efficacy in patients with OAB-UI ^45^. The effect size (from baseline) in both treatment groups compared with placebo was superior to either therapy alone ^45^. Only one phase IV trial, performed in Japan, confirmed that mirabegron was effective as an add-on therapy to anti-muscarinic treatment ^46^. This study (MILAI) assessed mirabegron (25 or 50 mg) with solifenacin (2.5 or 5 mg) over 16 weeks in 223 patients (at least 20 years old) with OAB. The authors reported significant improvements in mean number of UI or UUI episodes per 24 hours, OAB symptom score, and QOL-related scores in all treatment groups.

### Endocannabinoids

Early studies demonstrated that cannabis had therapeutic benefit on urgency incontinence in patients with multiple sclerosis. Constitutively active fatty acid amide hydrolase (FAAH) is the enzyme that breaks down endogenous cannabinoids which are released ‘on demand’ and can target cannabinoid and vanilloid receptors all of which play a role in bladder dysfunction. Thus, targeting FAAH (inhibitors/inactivators) may have a number of therapeutic applications, including treatment of bladder dysfunction and incontinence. A number of animal studies have shown that inhibition of FAAH has benefit in normalizing a number of urodynamic parameters in experiment bladder disorders (see review 47). Although pre-clinical studies have shown a potential benefit of FAAH inhibitors, recent evidence involving a phase I study in healthy human volunteers had revealed a number of off-target AEs showing that the drug was not safe to use in human studies ^48^. This also indicates a cautionary note whereby more extensive pre-clinical screening (including in human cells) for off-target effects may help to identify potential risks.

## Pharmacological management for stress incontinence

SUI is the involuntary leakage of urine on exertion, sneezing, or coughing ^49^. In clinical practice, the most efficacious approach to reduce the number of incontinence episodes is to increase musculature tone in the pelvic region (pelvic floor muscles) with exercise ^12, 50^. Although data supporting long-term use are limited, α1-AR antagonists have been used to increase urethral tone to ensure continued closure during episodes of increased pressure. Alternative drugs such as β2-AR agonists have been used in Japan for SUI treatment ^23^.

Studies report an increased risk of pelvic floor disorders after menopause and this may be linked to estrogen deficiency; thus, estrogen therapy may be beneficial in improving SUI in post-menopausal women. A meta-analysis of clinical trial data rated the evidence for efficacy of estrogen in reducing incontinence as low but well tolerated, much more so than alternative drugs for OAB/incontinence ^51, 52^. The route of administration appears to be very important as recently concluded by a Cochrane review. The authors reported worsening of UI with systemic estrogen versus an improvement in UI when estrogen was applied locally ^53^. Conversely, the addition of vaginal estrogen to IR or ER tolterodine did not improve urinary symptoms more than tolterodine alone ^51^. In addition, vaginal estrogen has been shown to normalize the microbiota and bladder function, making the case for preventive treatment ^54, 55^. Current guidelines suggest the use of estrogen as an adjunct therapy ^51^.

Duloxetine is a dual serotonin and noradrenaline reuptake inhibitor currently approved in Canada, the US, and Europe for depression. In 2004, duloxetine, under the trade name Yentreve, was approved and licensed in Europe as an add-on therapy for SUI ^56^. Despite reported efficacy in US and Canadian phase III trials ^57, 58^, licensing in the US and Canada failed because of concerns over incident suicidality. The FDA reported a doubling in suicide attempts in women receiving the drug for SUI compared with that in the general population (see product information at: https://www.fda.gov/downloads/drugs/developmentapprovalprocess/developmentresources/ucm328101.pdf). An independent analysis of the dataset used by the European Medicines Agency to license duloxetine was performed by a Canadian research group specifically assessing psychological disturbances. The authors concluded that the risks outweighed the benefits, thus raising concerns regarding the rationale for duloxetine use for treatment of SUI ^59, 60^. As of 2013, National Institute for Health and Care Excellence guidelines recommend duloxetine (Yentreve) as a second-line therapy in place of surgery for women with SUI, and counseling on expected AEs is advised ^61^.

## Conclusions

In terms of pharmacologic treatment for UI, many drugs exhibit poor efficacy and increased incidence of AEs. Alternative approaches such as the herbal medicine drug AOBO-001 have been introduced for OAB-incontinence and frequency in adults, and a phase II clinical trial is under way to study efficacy in adults with urge incontinence. There is a need for development of new therapeutic approaches, especially in terms of SUI, which have limited options. However, given the recent examples of off-target effects, one should carefully consider increased patient vulnerability to toxicity and AEs due to change in organ function which presents more opportunity for drug-disease interactions.
